# Tracheal Papilloma Mimicking Severe Asthma

**DOI:** 10.7759/cureus.46733

**Published:** 2023-10-09

**Authors:** Muzamil Khan, Khalil Diab

**Affiliations:** 1 Pulmonary Medicine, George Washington University School of Medicine and Health Sciences, Washington DC, USA

**Keywords:** tracheal masses, tracheal papillomatosis, cryotherapy, severe-asthma, tracheal papilloma

## Abstract

Tracheal papillomatosis is a rare and challenging condition characterized by the development of benign tumors in the trachea and bronchial tree. This case report presents a 53-year-old female with refractory papillomatosis and mediastinal lymphadenopathy. Despite three previous resections, the papilloma recurred, causing moderate tracheal narrowing and symptoms mimicking severe persistent asthma. In June 2023, the patient underwent bronchoscopy with successful tumor debulking cryosurgery, significantly improving her symptoms.

## Introduction

Tracheal masses present diagnostic challenges due to their diverse etiologies and clinical manifestations. The differential diagnosis for single tracheal masses encompasses a broad spectrum of conditions, including primary and metastatic malignancies, non-neoplastic lesions, and rare tumors. Squamous and adenoid cystic carcinoma are the most common primary tracheal malignancies, constituting approximately half of all cases. Additionally, bronchial carcinoids, mucoepidermoid carcinoma, and chondrosarcoma are among the trachea's primary neoplasms. Furthermore, non-neoplastic lesions, such as tuberculosis, inflammatory fibro-epithelial polyps, and mucus plugging, can mimic tracheal malignancies and should be considered in the differential diagnosis. In multiple tracheal masses, conditions like laryngotracheal papillomatosis, tracheal amyloidosis, and multifocal neoplasms should be carefully evaluated [1].

Tracheal papillomatosis (TP) is a benign condition characterized by papillomatosis growth in the tracheal/bronchial epithelium, primarily caused by human papillomavirus (HPV) infection. The two predominant HPV subtypes associated with TP are HPV-6 and HPV-11. The clinical presentation of TP is often nonspecific, varying from mild symptoms like cough to severe and potentially life-threatening conditions such as upper airway obstruction [2-4].

## Case presentation

We present a case of a 50-year-old female with severe persistent eosinophilic asthma. She had been a smoker for 15 years, smoked 0.5 packs per day, and used smokeless tobacco. The patient had moderate persistent asthma and was on prednisone (20 mg) - two tablets on the first day, one tablet on the second day, and a half tablet on the third day along with an albuterol inhaler, fluticasone-salmeterol inhaler, and tiotropium inhaler as needed till further follow-up. Due to her extensive 15-year smoking history and unresponsiveness to medication, a chest computed tomography (CT) scan was deemed necessary, which revealed a polypoidal endotracheal mass. Despite three previous resections of tracheal papilloma, the mass showed significant regrowth with moderate narrowing of the distal trachea and mediastinal lymphadenopathy.

**Figure 1:**
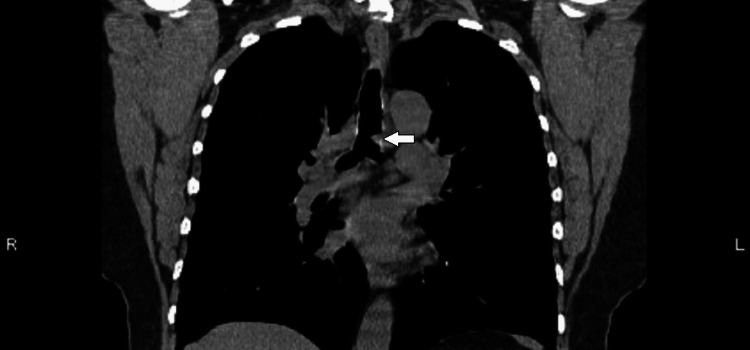
Coronal CT image with a white arrow indicating a solitary endotracheal polypoid mass near the bronchial bifurcation

**Figure 2:**
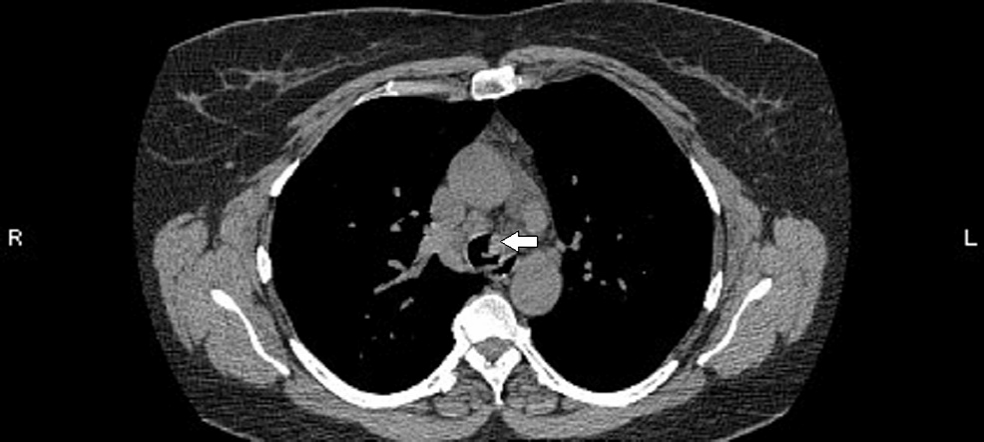
Axial CT scan with a white arrow demonstrating a possible squamous cell papilloma

**Figure 3:**
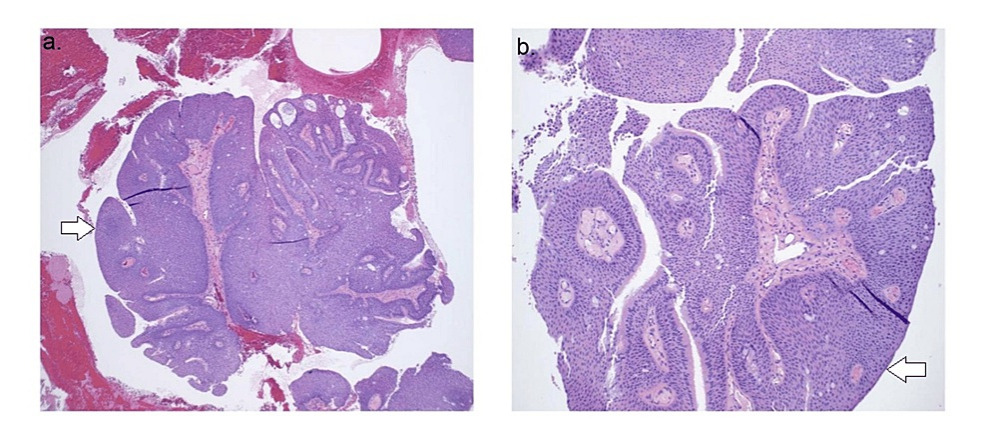
Low-power images: 4X (a) and 10X (b) with white arrows indicating a squamous papillomatous lesion

**Figure 4:**
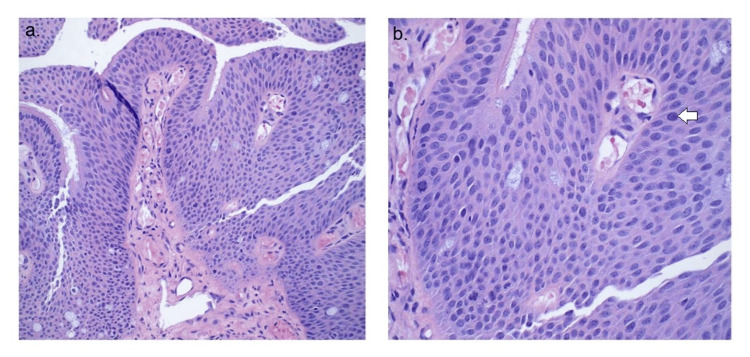
High magnification images: 20X (a) and 40X (b) showing mild to moderate dysplasia and focal mitotic figures. A white arrow in (b) demonstrates a mitotic figure.

The initial CT scan (Figure 1) on the patient revealed a solitary 1.3 cm endotracheal polypoid soft tissue in the lower anterior thoracic trachea, located near the bronchial bifurcation, causing mild narrowing of the trachea. The radiological features observed on the CT scan (Figure 2) indicated a squamous cell papilloma and a possible squamous cell malignant lesion. These findings warranted further investigation to determine the nature and extent of the tracheal mass. A biopsy was performed to obtain tissue samples from the endotracheal mass, and the histopathological examination revealed squamous papilloma with low to intermediate-grade dysplasia (Figures 3, 4). Following the initial diagnosis and treatment, close monitoring of the patient's condition was crucial to assess for any recurrence or progression of the tracheal papilloma. Subsequent CT scans were performed at scheduled intervals to evaluate the status of the mass. Unfortunately, during follow-up, recurrence of the mass was noticed multiple times in future CT scans, signifying the persistent nature of the condition.

The patient’s tracheal papillomatosis was initially managed through multiple resections performed by bronchoscopy with tumor debulking in the lower trachea using argon plasma electrocautery under general anesthesia. Despite these interventions, the papilloma exhibited a refractory nature, necessitating the consideration of an alternative treatment approach.

**Figure 5:**
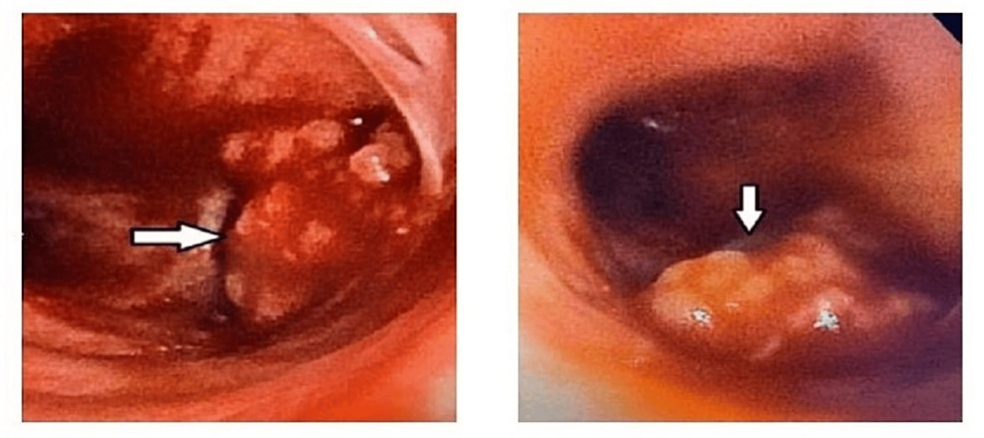
White arrows demonstrating a large endotracheal mass prior to the left mainstem bronchus that was visualized during EBUS EBUS: endobronchial ultrasound

Given the challenging and persistent nature of the tracheal papillomatosis, the decision was made to proceed with tumor debulking cryosurgery. Under general anesthesia, a regular bronchoscope was introduced through the endotracheal tube, allowing visualization of a large endotracheal mass situated before the left main stem takeoff (Figure 5). Utilizing a 2.4 mm cryoprobe, the mass was debulked with multiple passes, subjecting it to four 4-second freezing intervals without thawing. Excised tissue samples were collected and sent for pathology evaluation to confirm the diagnosis.

Following the initial debulking, an endobronchial ultrasound (EBUS) scope was employed to biopsy the mediastinal lymph nodes to assess the potential spread of the disease. Subsequently, the regular bronchoscope probes were reinserted, and cryoablation was performed to freeze the remaining area of the mass. Each freezing cycle consisted of 1 minute of freezing followed by 5 seconds of thawing, and this process was repeated four times to ensure adequate treatment.

The tumor-debulking cryosurgery was successful, leading to significant improvement in the patient’s symptoms. Tumor debulking cryosurgery provides a viable alternative for such cases, offering a minimally invasive approach to reduce tumor burden and alleviate airway obstruction. Post-procedure, the patient experienced relief from respiratory distress and reported better asthma control. No immediate complications, such as bleeding, were noted during or after the procedure.

## Discussion

Tracheal papillomatosis (TP), a manifestation of recurrent respiratory papillomatosis (RRP), can occur at any age, affecting both genders. It's more common in white individuals, with two age peaks: one in early childhood (average age 3.8 years) and another in middle age. Cases in the elderly are rare [4]. RRP leads to the growth of papillomas in the respiratory and upper gastrointestinal tracts, mainly in the larynx [5,6]. Most RRP cases involve the larynx and rarely appear as isolated TP [4].

Tracheal papillomatosis presents in two forms: juvenile-onset (JORRP) and adult-onset (AORRP). JORRP is believed to be transmitted from infected mothers to infants, but the exact method is unclear. Despite surgical removal, the disease often recurs, causing airway problems and voice changes, especially in children [6].

A case was reported by Chen YB et al., in which a 32-year-old hairdresser with a chronic cough for one year was initially misdiagnosed with asthma. Later, he presented with coughing up papillomatous tissue, revealing an endotracheal mass. Bronchoscopy and tissue removal relieved his symptoms, confirming tracheal papilloma with no malignancy [7].

Another case by Lam CW et al. involved a 52-year-old woman initially diagnosed with asthma and treated with steroids due to exertional breathlessness. She was later found to have an intratracheal lesion on a chest radiograph. Despite having no smoking or carcinogen exposure, she had stridor. Flexible bronchoscopy and chest CT revealed a cauliflower-like tumor obstructing the trachea. After surgical removal, the patient experienced immediate symptom relief and remained recurrence-free during 18 months of follow-up with regular bronchoscopic examinations [8].

Uysal A et al. reported a case in which a CT chest demonstrated multiple tracheal masses. There were no lesions suggestive of recurrent respiratory papillomatosis within the lung parenchyma. A bronchoscopy revealed several smooth papillomas arising in the trachea and extending to the right main stem airway. The largest papilloma caused an approximate 30% narrowing of the tracheal lumen. There were no papillomas in the oropharynx or vocal cords. Biopsies were obtained using electrocautery forceps to minimize bleeding. The biopsy was positive for benign squamous papilloma. There were no viral inclusions. The patient had a fiberoptic bronchoscopy via an endotracheal tube to remove the obstructing papillomas. Cryotherapy was used to destroy the tumors. The papillomas were soft and were easily frozen and destroyed with repeated cycles of cryotherapy. There was minimal bleeding. After the procedure, the patient immediately noted an improvement in his dyspnea [9].

Ko Y et al. reported an 82-year-old woman initially diagnosed with asthma but later found to have a large tracheal papilloma causing breathing problems. It was successfully treated with endoscopic ablation, and the condition did not return for two years [10].

A case by Yilidrim et al. involved a 40-year-old male smoker who had three months of progressive shortness of breath. A physical exam showed reduced lung sounds. Blood tests and the chest X-ray were normal. A chest CT revealed a near-obstructing mass in the distal trachea. Fiberoptic bronchoscopy removed the obstructive papillomatous lesion using mechanical debridement and cryotherapy. He received interferon alfa (IFN-α) for six months and remains under close follow-up with no recurrence in the eighth month [11].

This case study emphasizes the significance of distinguishing TP from asthma and other respiratory conditions to ensure appropriate treatment. It showcases how severe asthma-like symptoms initially led to mismanagement. Chest radiographs can initially present with normal findings; however, a chest CT scan can be considered, prompting further investigations and subsequent diagnosis of TP with endoscopy and biopsy [7]. Accurate diagnosis of TP is critical due to its potential to imitate asthma symptoms [7,10]. TP tends to recur despite successful resections, highlighting the need for more targeted treatment strategies. Multiple surgical resections were performed to address the papilloma and provide relief from symptoms. However, the recurrent behavior of the mass underscores the challenge of managing TP effectively. Tumor debulking cryosurgery, a minimally invasive approach, has shown promise in reducing tumor burden and alleviating airway obstruction in recurrent TP cases in which repeated resections via other measures have proven to be more invasive and less favorable. The case study also demonstrates the efficacy of cryosurgery in improving symptoms and asthma control [11]. To avoid misdiagnosis and delayed management, it is crucial to consider and rule out other conditions with similar symptoms to TP.

## Conclusions

Physicians should consider performing a chest CT scan when managing patients with persistent severe asthma, especially if it is unresponsive to conventional asthma treatment. This imaging modality can provide valuable insights into tracheal lesions and other potential underlying causes, aiding in accurate diagnosis and tailored treatment plans. Given the wide range of conditions that can present as tracheal lesions, it is essential to obtain biopsies from all suspicious lesions. Biopsies enable precise histological evaluation, facilitating the differentiation between benign and malignant entities and guiding appropriate management strategies. For patients diagnosed with recurrent tracheal papillomas, cryosurgery has demonstrated promising results as a feasible and minimally invasive treatment option. Compared to other measures, cryosurgery offers potential benefits, such as reduced morbidity and improved patient outcomes, making it an attractive therapeutic approach. Tracheal papillomatosis is difficult to treat, usually recurrent, and needs close observation after treatment.

## References

[1] Gaillard F, Bell D, Di Muzio B (2021). Tracheal masses. Radiopedia.

[2] Harris K, Chalhoub M (2011). Tracheal papillomatosis: what do we know so far?. Chron Respir Dis.

[3] Valentino J, Brame CB, Studtmann KE, Manaligod JM (2002). Primary tracheal papillomatosis presenting as reactive airway disease. Otolaryngol Head Neck Surg.

[4] David PB, Owen L (2007). Isolated tracheal papillomatosis—an infrequent cause of chronic cough. Respir Med.

[5] Armstrong LR, Derkay CS, Reeves WC (1999). Initial results from the national registry for juvenile-onset recurrent respiratory papillomatosis. Arch Otolaryngol Head Neck Surg.

[6] Mounts P, Shah KV (1984). Respiratory papillomatosis: etiological relation to genital tract papillomaviruses. Prog Med Virol.

[7] Chen YB, Jiang JH, Guo LC, Huang JA (2016). Primary tracheal papilloma disguised as asthma: A case report. J Asthma.

[8] Lam CW, Talbot AR, Yeh KT, Lin SC, Hsieh CE, Fang HY (2004). Human papillomavirus and squamous cell carcinoma in a solitary tracheal papilloma. Ann Thorac Surg.

[9] Uysal A, Milligan S, Owens M, Wellikoff A, Liendo C, Trinh C (2013). Multiple tracheal squamous papillomas in an adult treated with cryotherapy. Chest.

[10] Ko Y, Kim C, Park YB (2020). Solitary tracheal papilloma. Am J Respir Crit Care Med.

[11] Yilidrim F, Türk M, Demircan S, Akyürek N, Yurdakul AS (2015). Tracheal papilloma treated with cryotherapy and interferon-α: a case report and review of the literature. Case Rep Pulmonol.

